# Optically initialized robust valley-polarized holes in monolayer WSe_2_

**DOI:** 10.1038/ncomms9963

**Published:** 2015-11-25

**Authors:** Wei-Ting Hsu, Yen-Lun Chen, Chiang-Hsiao Chen, Pang-Shiuan Liu, Tuo-Hung Hou, Lain-Jong Li, Wen-Hao Chang

**Affiliations:** 1 Department of Electrophysics, National Chiao Tung University, Hsinchu 30010, Taiwan; 2 Institute of Atomic and Molecular Sciences, Academia Sinica, Taipei 10617, Taiwan; 3 Department of Automatic Control Engineering, Feng Chia University, Taichung 40724, Taiwan; 4 Department of Electronics Engineering and Institute of Electronics, National Chiao Tung University, Hsinchu 30010, Taiwan; 5 Taiwan Consortium of Emergent Crystalline Materials (TCECM), Ministry of Science and Technology, Taipei 10622, Taiwan; 6 Physical Sciences and Engineering Division, King Abdullah University of Science and Technology, Thuwal 23955-6900, Kingdom of Saudi Arabia

## Abstract

A robust valley polarization is a key prerequisite for exploiting valley pseudospin to carry information in next-generation electronics and optoelectronics. Although monolayer transition metal dichalcogenides with inherent spin–valley coupling offer a unique platform to develop such valleytronic devices, the anticipated long-lived valley pseudospin has not been observed yet. Here we demonstrate that robust valley-polarized holes in monolayer WSe_2_ can be initialized by optical pumping. Using time-resolved Kerr rotation spectroscopy, we observe a long-lived valley polarization for positive trion with a lifetime approaching 1 ns at low temperatures, which is much longer than the trion recombination lifetime (∼10–20 ps). The long-lived valley polarization arises from the transfer of valley pseudospin from photocarriers to resident holes in a specific valley. The optically initialized valley pseudospin of holes remains robust even at room temperature, which opens up the possibility to realize room-temperature valleytronics based on transition metal dichalcogenides.

## Abstract (web summary)

The long associated lifetimes required to exploit valley polarization in electronic devices, along with charge and spin, have yet to be observed. Here, the authors demonstrate the photoexcitation of long-lived valley-polarized holes in WSe_2_.

## Graphical abstract

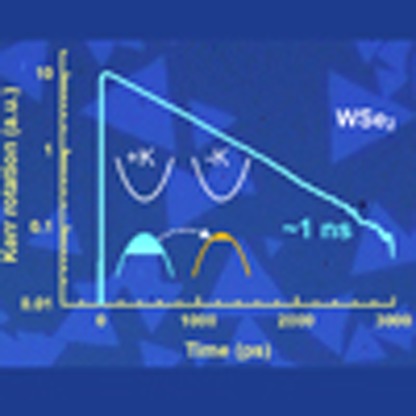

The valley pseudospin in addition to charge and spin of electrons in materials has been considered as a new degree of freedom for information processing in next-generation electronic and optoelectronic devices^1^,^2^,^3^,^4^. Monolayer transition metal dichalcogenides (TMDs), which possess two inequivalent but energy-degenerate valleys (±K points) at the corners of the hexagonal Brillouin zone^3^,^4^,^5^, emerge as a promising candidate to realize such valleytronic devices. Monolayer TMDs further feature their coupled spin and valley physics stemming from the inherent inversion symmetry breaking in the honeycomb lattice structure and the strong spin–orbit coupling in the d-orbits of the transition metal, leading to strong valley-contrasting optical selection rules at ±K valleys^3^,^4^,^5^ (Fig. 1a). This unique property provides an unprecedented opportunity to generate, control and detect valley polarization (that is, the population imbalance between ±K valleys) by optical means. Indeed, a number of valley-contrasting phenomena, including exciton valley polarization^6^,^7^ and valley coherence^8^, valley Hall effect^9^, valley-selective optical Stark effect^10^,^11^ and valley Zeeman effect^12^,^13^,^14^,^15^ have been demonstrated recently. These observations demonstrated the feasibility of exploiting the valley pseudospin in TMDs for future valleytronics.

Despite the success in manipulating valley polarizations in TMDs, knowledge about the valley-depolarization mechanism and the timescale of valley lifetime, which determines device operations and information storages, is still very limited. Recently, a number of time-resolved measurements on valley dynamics in various TMDs have been reported^16^,^17^,^18^,^19^,^20^,^21^,^22^,^23^. Although the valley polarization in monolayer TMDs is expected to be very robust due to the valley-contrasting spin splitting and the large valley separation in momentum space^24^,^25^, direct experimental evidences for the anticipated long-lived valley polarization remain absent. In addition, valley pseudospin of electrons or holes is expected to be a more robust information carrier compared with short-lived excitons. In III–V and II–VI semiconductors, spin polarization of resident carriers can be optically initialized through spin transfer from photogenerated carriers^26^,^27^. However, initializing valley pseudospin of single carrier species (electrons or hole) in TMD has yet to be demonstrated.

Here we demonstrate a valley-selective optical pumping scheme to create long-lived valley-polarized holes in monolayer WSe_2_. We used time-resolved Kerr rotation (TRKR) spectroscopy (Fig. 1a), which gives a direct measurement of the population imbalance between valleys induced by helical light. Since the Kerr rotation measured at normal incidence is also sensitive to the net magnetization projection along the surface normal, this technique is very ideal for detecting the net spin and valley pseudospin even after carrier recombination. On the other hand, monolayer WSe_2_ with a very large spin–orbit coupling (∼0.46 eV), in contrast to MoS_2_ (∼0.15 eV)^28^, provides an excellent test bed to observe long-lived valley polarization. The WSe_2_ monolayers investigated here were grown by chemical vapour deposition (CVD) on sapphire substrates^29^ (Fig. 1b). The sample was studied at *T=*10 K, unless otherwise specified in temperature-dependent measurements.

## Results

### Exciton valley polarization

**Figure 1:**
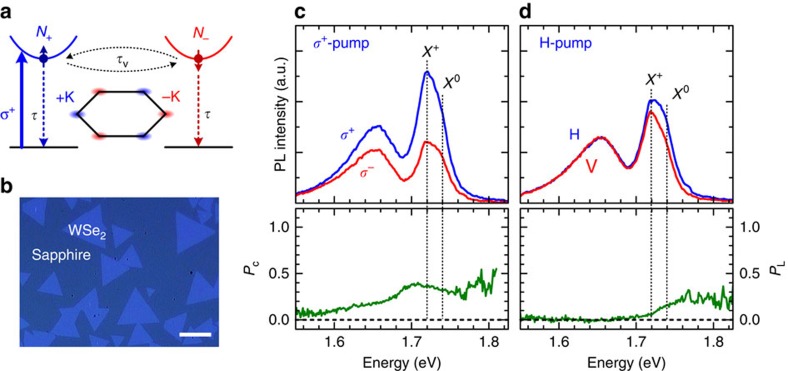
Valley polarization and coherence in monolayer WSe_2_. (**a**) Schematic of the valley-dependent exciton dynamics. The steady-state exciton population *N*_+_ (*N*_−_) in the +K (−K) valley under σ^+^ excitation is determined by the valley lifetime *τ*_v_ and the exciton lifetime *τ*. (**b**) Optical microscopy image for CVD-grown monolayer WSe_2_ triangles on sapphire substrate. Scale bar, 10 μm. (**c**) Valley polarization. The upper panel shows the σ^+^ (blue curve) and σ^−^ (red curve) components of circular-polarization-resolved PL spectra under σ^+^ laser excitations. The lower panel shows the degree of circular polarization (*P*_C_). (**d**) Valley coherence. The upper panel shows the H (blue curve) and V(red curve) components of linear-polarization-resolved PL spectra under H-polarized laser excitations, where H (V) stands for horizontally (vertically) linear polarization. The lower panel shows the degree of linear polarization (*P*_L_). The emission peaks of neutral exciton (X^0^) and positive trion (X^+^) are indicated by vertical dotted lines.

We first identify the exciton species and characterize the valley polarization in monolayer WSe_2_ by polarization-resolved photoluminescence (PL) measurements. Figure 1c shows the PL spectra excited by right circularly polarized (σ^+^) laser light at 1.96 eV. The peak at 1.72 eV and a shoulder near 1.74 eV are attributed to the emissions from positive trion (X^+^) and neutral exciton (X^0^) at K valleys, respectively. The dominant X^+^ emission arises from the unintentionally p-type doping in our CVD-grown WSe_2_ on sapphire substrates, as observed in transport measurements (Supplementary Fig. 1). We have also measured the gate-dependent PL spectra for the CVD-grown WSe_2_ monolayers on a back-gate device (Supplementary Fig. 2). The PL peaks of X^0^, X^+^ and negative trion (X^−^) can be identified by monitoring the PL spectrum as a function of gate voltage. The binding energy of X^+^ (X^−^) is found to be ∼20±2 meV (∼29±2 meV), consistent with previous PL studies on monolayer WSe_2_ (ref. 8). The broad PL band near 1.66 eV probably arises from localized excitons (LX) bound to defects or impurities. The σ^+^ laser excitation leads to a sizable PL circular polarization (*P*_C_∼0.35) around X^0^ and X^+^ peaks (Fig. 1c), which is a clear signature of exciton valley polarization^5^,^6^,^7^,^8^. We have also examined the valley coherence by linear-polarization-resolved PL measurements (Fig. 1d). According to previous PL studies on WSe_2_ (ref. 8), only X^0^ can emit linearly polarized PL in accordance with the polarization of excitation laser. The measurements of valley coherence thus provide an alternative way to distinguish the exciton and trion peaks, especially when they are not clearly spectrally resolved. As shown in Fig. 1d, a sizable PL linear polarization (*P*_L_∼0.18) can be observed only in the vicinity of X^0^ emission, while emissions from X^+^ and LX are unpolarized. The spectral dependence of valley coherence further confirms our assignments for X^0^ and X^+^ emissions.

The degree of exciton valley polarization is determined by two timescales: the valley lifetime *τ*_v_ and the exciton lifetime *τ*, which is related to the radiative lifetime *τ*_r_ and non-radiative lifetime *τ*_nr_ by 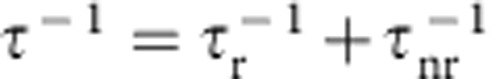. From standard rate equations for exciton generation and recombination, the steady-state PL polarization is given by *P*_C_=*P*_0_/(1+*τ*/*τ*_v_), where *P*_0_ is the initial circular polarization, which could be less than unity due to the initial intervalley scatterings caused by defects, impurities or the substrate effects. In the past few years, high-PL circular polarizations have been observed in various TMD monolayers^5^,^6^,^7^,^8^. It is therefore a common belief that the valley lifetime is very long compared with the exciton lifetime, that is, 
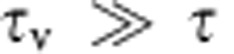
. For example, in ref. 7, a hole valley lifetime of >1 ns was predicted based on the observed near-unity *P*_C_ and an estimated exciton lifetime (∼50 ps) in MoS_2_. However, according to recent time-resolved studies^16^,^17^,^18^,^19^,^20^,^21^,^22^,^23^ on various TMDs, the observed valley lifetimes are very short; typically<10 ps. To date, direct experimental evidence for the anticipated long-lived valley polarization remain absent.

### Time-resolved Kerr rotation measurements

**Figure 2:**
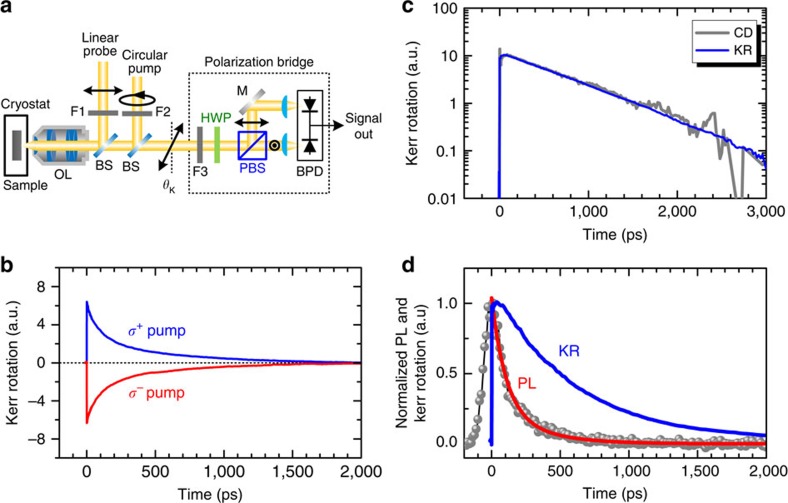
Time-resolved Kerr rotation (TRKR) measurements. (**a**) Schematic of the experimental set-up for TRKR spectroscopy. The circularly polarized pump pulses and the linearly polarized probe pulses with a time delay were focused through an objective lens (OL) and at normal incidence to the sample surface. The wavelengths of the pump and the probe pulses were selected individually by two edge-pass filters F1 and F2. The Kerr rotation (*θ*_K_) of the reflected probe pulses was analysed by a polarization bridge consisting of a half waveplate (HWP), a polarization beamsplitter (PBS) and a pair of balanced photodiodes (BPD). Before entering the polarization bridge, the pump beam was filtered out by a long-pass filter F3. BS stands for beamsplitter and M stands for mirror. (**b**) TRKR traces probed at 1.719 eV under σ^+^ (blue) and σ^−^ (red) pumping. The Kerr rotation signal changes sign when reversing the pump pulse helicity. (**c**) A comparison between the measured Kerr rotation (KR, blue curve) and circular dichroism (CD, grey curve) obtained from helicity-resolved transient reflection using the same experimental conditions. Both KR and CD show the same decay dynamics. (**d**) Temporal evolutions of PL intensity (grey symbols) and KR (blue curve). The red curve is a single-exponential fit to the PL decay.

To study valley lifetime, we performed two-colour TRKR measurements (Fig. 2a and Methods) on the monolayer WSe_2_. In TRKR experiments, the circularly polarized pump pulse generates valley-polarized excitons in a specific valley. The temporal evolution of the population imbalance was then monitored by the Kerr rotation *θ*_K_(*t*) of the reflected linearly polarized probe pulse with a time delay *t*. In Fig. 2b, we show the TRKR traces probed at 1.719 eV under σ^+^ and σ^−^ laser excitations. A reversal in sign of the Kerr signal is observed when reversing the pump pulse helicity, indicating that the Kerr signal is originated from the optically initialized valley polarization. The temporal decay of Kerr signal thus reflects the valley depolarization. This can be further verified by the helicity-resolved transient reflection using circularly polarized pump and probe pulses (Supplementary Fig. 3). We found that the reflection changes probed by σ^+^ and σ^−^ pulses show a strong circular dichroism. As shown in Fig. 2c, the measured *θ*_K_(*t*) reproduces the time evolution of circular dichroism very well, confirming that the Kerr signal is a measure of the population imbalance between ±K valleys.

The most significant finding is that the Kerr rotation *θ*_K_(*t*) exhibits a single-exponential decay with a very long lifetime (∼700 ps for the case shown in Fig. 2c). Measurements on different monolayer flakes show that the Kerr signal lifetime *τ*_K_ is in the range of 600–700 ps at *T*=10 K, and only varies slightly from one to another (Supplementary Fig. 4). The long-lived valley polarization is about 1–2 orders of magnitude longer than the typical emission lifetime (∼4–18 ps) in WSe_2_ reported in literatures^19^. We have measured time-resolved PL to study the emission lifetime in our CVD-grown WSe_2_ (Fig. 2d). Although we are unable to extract the emission lifetimes of exciton and trion from the PL decay due to the temporal resolution limit (∼100 ps) of our system, it can be seen clearly that the Kerr signal persists after the PL decays completely. This observation indicates that the Kerr signal is not induced by exciton valley polarization, since the exciton population imbalance will vanish after recombination. This can be understood from a simple rate-equation analysis (Supplementary Note 1). Considering the temporal evolution of exciton population imbalance 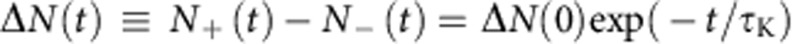, where *N*_±_ is the exciton population in the ±K valleys and *τ*_K_ is the decay time of the population imbalance, we obtain 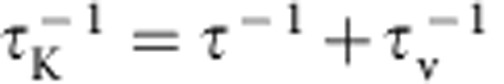. If the valley lifetime is very long compared with the exciton recombination lifetime 
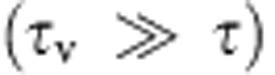
, we have *τ*_K_≈*τ*. In general, the measured *τ*_K_ will never exceed *τ* for pure exciton valley polarization.

### Transfer of valley pseudospin to resident holes

**Figure 3:**
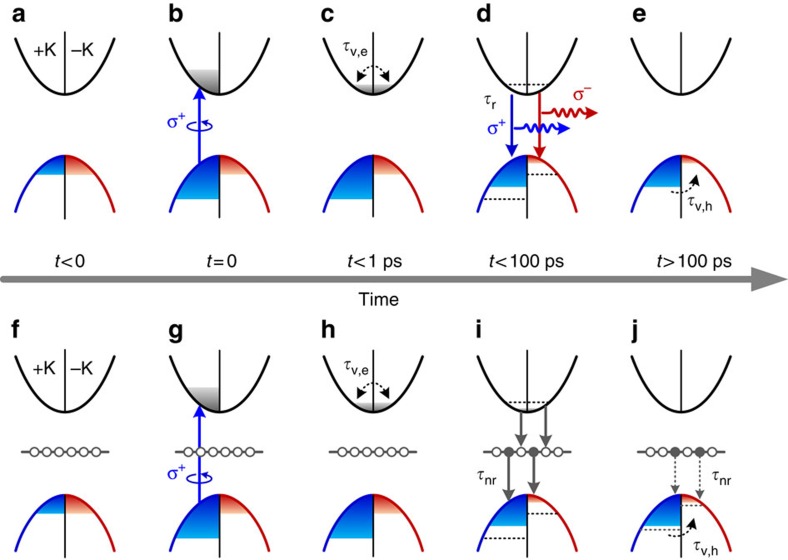
Optically initialized valley-polarized holes. Schematics illustrating the processes of valley pseudospin transfer from photocarriers to resident holes through (**a**–**e**) radiative and (**f**–**j**) defect-assisted non-radiative recombination in different temporal regions. (**a**,**f**) The resident holes are equally occupied in +K and -K valleys before optical excitations (*t*<0). The defect levels in **f** are fully empty (open circles) at equilibrium. (**b**,**g**) After optical pumping by σ^+^ light (up arrow) at *t*=0, electrons and holes are injected into +K valley. (**c**,**h**) The photogenerated electrons get unpolarized first within the timescale of *t*<1 ps. *τ*_v,e_ denotes the electron valley lifetime. (**d**,**i**) Recombination (down arrows) occurs in both valleys through radiative (**d**) and defect-assisted processes (**i**) within the timescale of *t*<100 ps. *τ*_r_ and *τ*_nr_ denote the radiative and non-radiative recombination lifetimes, respectively. (**e**,**j**) A net valley polarization of holes in the +K valley is created and then depolarized through intervalley scatterings in the timescale of *t*>100 ps. *τ*_v,h_ denotes the hole valley lifetime. If some electrons remain trapped in defect levels in this timescale, the hole populations in both valleys will be affected by the slow non-radiative recombination.

The long-lived Kerr signal can be explained by the transfer of valley pseudospin from photocarriers to the resident carriers, just as the commonly observed spin polarization transfer to resident electrons in n-doped III–V or II–VI semiconductors^26^,^27^. In our CVD-grown WSe_2_ monolayers, the transfer of valley pseudospin can be mediated by positive trion X^+^, leaving behind valley-polarized holes after trion recombination. The processes are illustrated in Fig. 3a–e. Initially, the resident holes are equally occupied in both valleys (Fig. 3a). Under circularly polarized (σ^+^) light excitations, excitons are injected into a specific valley (+K), giving rise to exciton valley polarization (Fig. 3b). If the valley lifetimes of electron (*τ*_v,e_) and hole (*τ*_v,h_) are long compared with the exciton lifetime *τ*, excitons will recombine mostly in the same valley (+K) and hence leave the spin and valley indices of the resident holes unchanged. The transfer of exciton valley pseudospin to the resident holes can occur when the valley lifetimes of the photogenerated electrons and holes have very different timescales and satisfy *τ*_v,e_<*τ*<*τ*_v,h_. Indeed, recent transient absorption measurements for monolayer MoS_2_ (ref. 20) and WS_2_ (ref. 21) revealed that the electron valley lifetime is surprisingly short (*τ*_v,e_<1 ps), while the hole valley lifetime is expected to be longer. This means that photogenerated electrons will get unpolarized first (Fig. 3c), and then recombine with resident holes in the opposite valley (−K), giving rise to a net valley polarization of holes in the same valley (+K) (Fig. 3d). The Kerr signal decay thus reflects the single-carrier valley depolarization of holes (Fig. 3e) and the decay time gives a direct measurement of hole valley lifetime *τ*_K_=*τ*_v,h_. We have also developed a simple rate-equation model considering separate depolarization of valley electrons and holes (Supplementary Note 1; Supplementary Fig. 5). The calculated temporal evolutions of the valley carriers also show that a pure valley hole population imbalance is created after carrier recombination.

Apart from radiative recombination, the photogenerated carriers in TMD can also recombine non-radiatively. Indeed, the low quantum efficiencies in typical TMD emissions certainly imply that non-radiative recombination is significant. This is particularly true for the CVD-grown TMDs, which contain appreciable amounts of defects as compared with those exfoliated from a natural bulk crystal. Therefore, the transfer of valley pseudospin from the photogenerated carriers to the resident holes can also occur through defect-assisted recombination processes, as shown in Fig. 3f–j. In the p-type sample, the defect levels are presumably fully empty (or fully occupied by holes) under equilibrium at low temperatures (Fig. 3f). After optical pumping, photogenerated electrons, followed by holes, are captured by the defect levels (Fig. 3i). Such defect-assisted recombination could be very efficient via Auger capture processes, as indicated by recent time-resolved measurements^30^ and theoretical study^31^ on MoS_2_. Therefore, we expect that the majority of photogenerated carriers have decayed through this channel within the timescale <100 ps, according to our time-resolved PL (Fig. 2d) and pump–probe transient reflection measurements (Supplementary Fig. 6). On the other hand, our transient reflection data further suggest the presence of some defect traps that undergo very slow (∼1 ns) non-radiative recombination in our sample. As shown in Fig. 3j, the slow defect-assisted recombination will affect the hole populations in both valleys, but with different rates proportional to the valley hole population. In this case, the slow defect-assisted recombination will contribute to the valley hole depolarization in addition to the intrinsic intervalley scattering.

### Probe energy dependence

**Figure 4:**
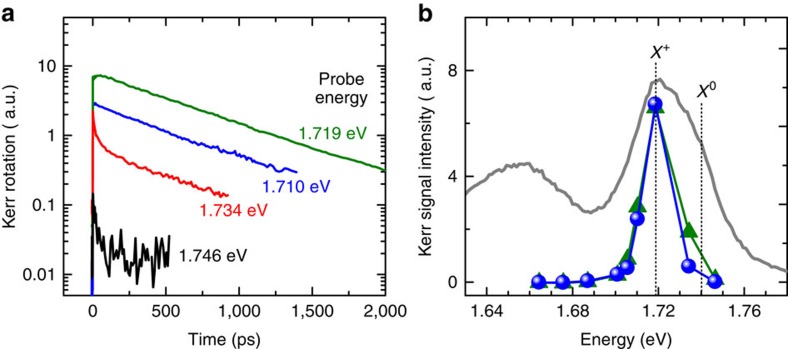
Probe energy dependence of TRKR. (**a**) TRKR traces measured at various probe energies. The pump energy is always ∼12 meV higher than the probe energy. (**b**) The Kerr signal intensities at delay times *t*=2.5 ps (green, solid triangles) and *t*=100 ps (blue, solid circles) as a function of probe energy. The PL spectrum is also included (grey curves) for comparison. The emission peaks of neutral exciton (X^0^) and positive trion (X^+^) are indicated by vertical dotted lines. The Kerr signal exhibits a maximum when the probe energy is on resonance with the X^+^ peak. As the probe energy was tuned close to the X^0^ peak, the Kerr signals become weak and very short-lived.

The transfer of valley pseudospin can be further examined by TRKR measurements at different probe energies, as shown in Fig. 4a,b. In the experiments, both the pump and probe energies are varied while the pump energy is always ∼12 meV higher than the probe (see Methods). The Kerr signal exhibits a maximum when the probe energy is on resonance with the X^+^ peak at 1.719 eV (Supplementary Fig. 7; Supplementary Note 2). As the probe energy was tuned close to X^0^ peak, the Kerr signal decreases significantly. In addition, the Kerr signal probed near X^0^ peak is very short-lived and is significant only for *t*<10 ps (Fig. 4b). From the rapid initial decay probed at 1.734 eV, we deduce a valley lifetime of ∼10 ps for X^0^. This value is consistent with ref. 23, where the valley lifetime for X^0^ is found to be very short (*τ*_v_=6 ps) at low temperatures (*T*=4–30 K), and even shorter at elevated temperatures. The short valley lifetime for X^0^ has been attributed to the strong electron–hole Coulomb exchange interaction, which efficiently switches the spin and valley indices of both the electron and hole of an exciton between ±K valleys in picosecond ranges. This means that X^0^ would depolarize within a timescale faster than (or comparable to) the recombination lifetime, and hence the formation of long-lived valley-polarized holes is suppressed. On the other hand, when the probe energy is tuned to the X^+^ resonance at 1.719 eV, the pump energy (∼1.731 eV) is below the X^0^ resonance. The pump pulses thus directly generate valley-polarized trions, which are free from electron–hole Coulomb exchange, and hence can last the valley polarization after recombination. This also highlights the important role played by X^+^ in the transfer of valley pseudospin to the resident holes. It is worth to mention that when probing the Kerr signals near the X^0^ resonance, the pump pulses also generate X^+^ in addition to X^0^. The absence of a long-lived Kerr signal probed at 1.745 eV implies that the trion valley polarization might also depolarize rapidly due to the coexistence of X^0^ and X^+^, as observed in ref. 19.

### Temperature-induced valley depolarization

**Figure 5:**
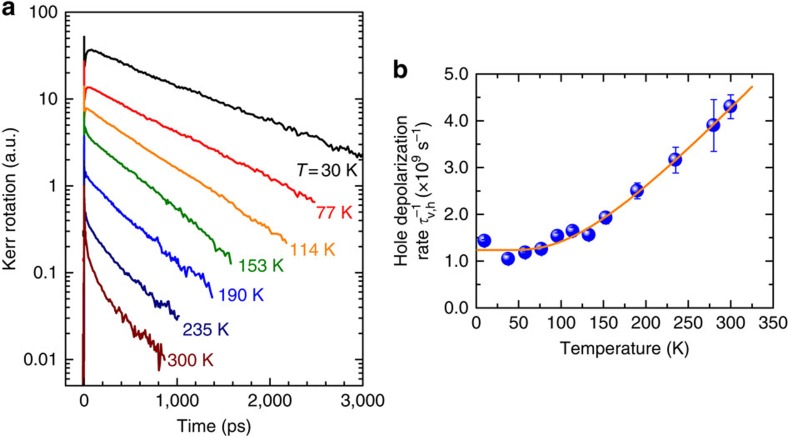
Temperature dependence of hole valley depolarization. (**a**) TRKR traces measured at various temperatures. (**b**) The valley-depolarization rate of the hole 
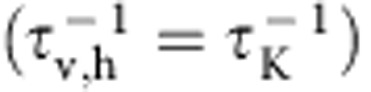
 as a function of temperature. The solid line is the fitting curve considering depolarization through phonon-mediated intervalley scatterings. Increasing the temperature only leads to a moderate increase in the valley-depolarization rate. At room temperature (*T*=300 K), the hole valley lifetime is found to be ∼230 ps, which is still at least an order of magnitude longer than the recombination lifetime. The error bars in **b** are estimated from the uncertainties of exponential fittings.

We have measured TRKR at elevated temperatures, as shown in Fig. 5a. In the experiments, the probe energy is tuned to on resonance with the trion peak according to temperature-dependent PL spectra. An even longer Kerr lifetime up to *τ*_K_=950 ps was observed at *T=*30 K, which is probably caused by the reduced concentration of resident holes at elevated temperatures. From the measured Kerr lifetimes, we deduced the valley-depolarization rate of the hole 
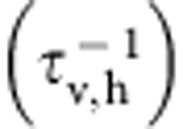
 as a function of temperature (Fig. 5b). Increasing the temperature only leads to a moderate increase in the valley-depolarization rate. The valley depolarization at elevated temperatures is presumably caused by phonon-mediated intervalley scatterings. By fitting the temperature dependence to 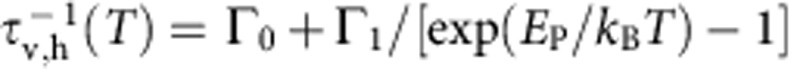, where *E*_P_ is the involved phonon energy, we obtained *E*_P_=32 meV=259 cm^−1^. Interestingly, the deduced *E*_P_ is very close to the phonon energy of the out-of-plane vibration modes (∼250 cm^−1^) near the K valley of monolayer WSe_2_ (ref. 32). This implies that the zone edge phonons play an important role in the hole intervalley scatterings. Recent theoretical studies suggest that the participated phonon modes could be influenced by the presence of substrate^33^,^34^. Further investigations are necessary to clarify the underlying mechanisms. It is noteworthy that the hole valley pseudospin remains robust even at room temperature (*T*=300 K), with a valley lifetime up to *τ*_v,h_∼230 ps, which is still at least an order of magnitude longer than the recombination lifetime.

## Discussion

In summary, we demonstrate, for the first time, a valley-selective optical pumping scheme to create long-lived valley-polarized holes in monolayer WSe_2_. Using TRKR spectroscopy, which directly measures the population imbalance between valleys induced by helical light, we observe a long-lived valley polarization for positive trion with a lifetime approaching ∼1 ns, which is much longer than the trion lifetime (∼10–20 ps). This observation provides an experimental evidence of the valley pseudospin transfer from valley-polarized photocarriers to resident holes in a specific valley. Compared with much short-lived excitons, the long-lived valley-polarized single carrier species in TMDs offer a unique platform to explore valleytronic applications. More importantly, we found that the valley-polarized holes remain robust even at room temperature, with a valley lifetime only reduced by a factor of ∼3–4 compared with that at low temperatures. We believe that it is possible to improve the valley lifetime by further optimizing the crystalline quality or by isolating the substrate effect. The long-lived and robust valley-polarized holes open up the opportunity to realize room-temperature valleytronics based on TMD.

## Methods

### Growth of monolayer WSe_2_

High-crystalline-quality monolayer WSe_2_ were synthesized on sapphire substrates by CVD in a horizontal hot-wall chamber. High-purity WO_3_ (99%, Alfa) and Se powders (99.5%, Alfa) were used as initial reactants. The centre heating zone with the WO_3_ powders was heated to 925 °C, while the Se powders placed at the upper stream side was maintained at 270 °C. The WSe_2_ samples were obtained by placing the substrate at the downstream side using an Ar/H_2_ flowing gas at 1 Torr for 15 min. The detailed growth procedures can be found in ref. 29.

### Optical measurements

The PL and TRKR spectroscopies were conducted in a home-made optical microscope using a × 50 objective lens (numerical aperture=0.42) to focus laser beams onto the sample surface. The sample was cooled down to *T*=10 K in a cryogen-free low-vibration cryostat. For PL measurements, a He-Ne laser (633 nm) was used as the excitation source. The typical excitation power is 200 μW. The PL signals were collected by the same objective lens, analysed by a 0.75-m monochromator and detected by a nitrogen-cooled charge-coupled device camera. The PL polarizations were analysed by the combination of half/quarter waveplates and a polarizer in front of the monochromator. For time-resolved PL measurements, the laser source was a 635-nm pulsed laser diode (∼90 ps, 40 MHz) and the PL signals were detected by a fast avalanche diode with a timing jitter of ∼50 ps. The PL decay traces were recorded by the time-correlated single-photon counting technique with a temporal resolution of ∼100 ps.

For TRKR measurements, the laser beam from a mode-locked Ti:sapphire laser (∼150 fs, 80 MHz) was split into a pump beam and a probe beam (Fig. 2a). Both the pump and probe beams were focused to a spot size of ∼2.5 μm through the objective lens and at normal incidence to the sample surface. The wavelengths of the pump and the probe pulses were selected individually by two edge-pass filters. A short-edge-pass filter was used for the pump pulse, while a long-edge-pass filter was used for the probe pulse. In our experiments, we first tune the central wavelength of the Ti:sapphire laser and then carefully adjust the tilted angles of the two filters, such that the pump energy is always ∼12 meV higher than the probe (Supplementary Fig. 8). The pump beam was circularly polarized by using a quarter waveplate, while the probe beam was linearly polarized through a polarizer. The time delay of the probe pulses was controlled by a mechanical stage. The Kerr rotation of the reflected probe pulses as a function of delay time was analysed by a polarization bridge using the balanced detection scheme. Before entering the polarization bridge, the pump beam was filtered out by a long-pass filter. The pump beam was modulated by a mechanical chopper at ∼2 kHz for lock-in detection.

The time-averaged powers of the pump and probe beams for the data shown in the main text were 500 and 100 μW, respectively. According to our Kerr measurements with various pump and probe powers at 300 and 10 K, the decay dynamics of the long-lived Kerr signal does not show significant change for pump powers ranging from 50 to 500 μW (Supplementary Fig. 9). We intentionally adapted a higher pump power (500 μW) to improve the signal-to-noise ratio of those weaker Kerr signals probed at an energy detuned from X^+^ resonance or at elevated temperatures, such that the same experimental condition can be maintained in the energy-dependent and temperature-dependent measurements.

## Additional information

**How to cite this article:** Hsu, W.-T. *et al*. Optically initialized robust valley-polarized holes in monolayer WSe_2_. *Nat. Commun.* 6:8963 doi: 10.1038/ncomms9963 (2015).

## Supplementary Material

Supplementary Information Supplementary Figures 1-9, Supplementary Notes 1-2 and Supplementary References.
