# Corrigendum: Optically initialized robust valley-polarized holes in monolayer WSe_2_

**DOI:** 10.1038/ncomms10396

**Published:** 2016-02-01

**Authors:** Wei-Ting Hsu, Yen-Lun Chen, Chang-Hsiao Chen, Pang-Shiuan Liu, Tuo-Hung Hou, Lain-Jong Li, Wen-Hao Chang

Nature Communications 6: Article number: 8963 10.1038/ncomms9963 (2015); Published: 11252015; Updated: 02012016

The original version of this Article contained a typographical error in the spelling of the author Chang-Hsiao Chen, which was incorrectly given as Chiang-Hsiao Chen. This has now been corrected in both the PDF and HTML versions of the Article.

