# Supplementary material for: Optically initialized robust valley-polarized holes in monolayer WSe2
Source: Nat Commun. 2015 Nov 25;6:8963. doi: 10.1038/ncomms9963 (PMC4674763; doi:10.1038/ncomms9963)
Supplement: Supplementary Information — Supplementary Figures 1-9, Supplementary Notes 1-2 and Supplementary References. [file ncomms9963-s1.pdf]

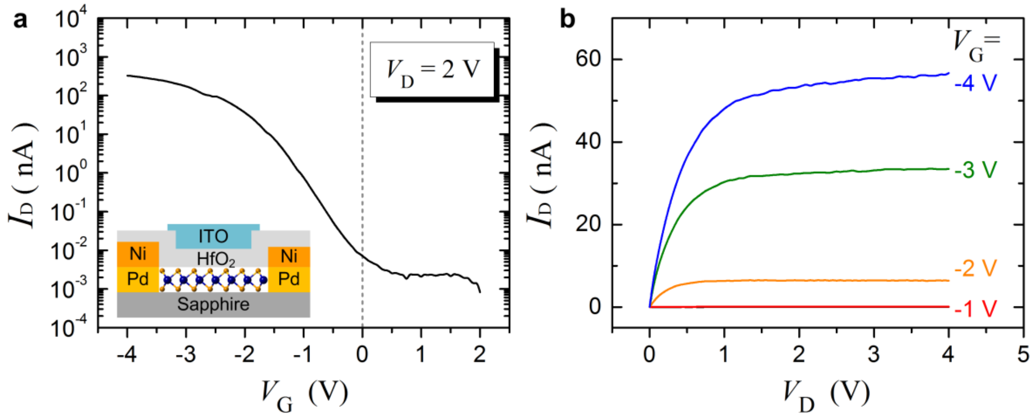

Supplementary Figure 1 | Device characteristics of a top-gate field-effect transistor (FET) fabricated on the as-grown WSe_2/sapphire. (**a**) The typical transfer curve ($I_D - V_G$) and (**b**) the output ($I_D - V_D$) characteristics of the top-gate WSe_2 FET. Inset: Schematic of the device structure. The top-gate FET is fabricated on an as-grown WSe_2/sapphire sample using Ni(25 nm)/Pd(15 nm) as the source/drain metals, and a 25-nm HfO_2 gate dielectric with indium tin oxide (ITO) on top as the gate contact (inset). The transfer curve shows that the WSe_2 FET exhibits *p*-type characteristic.

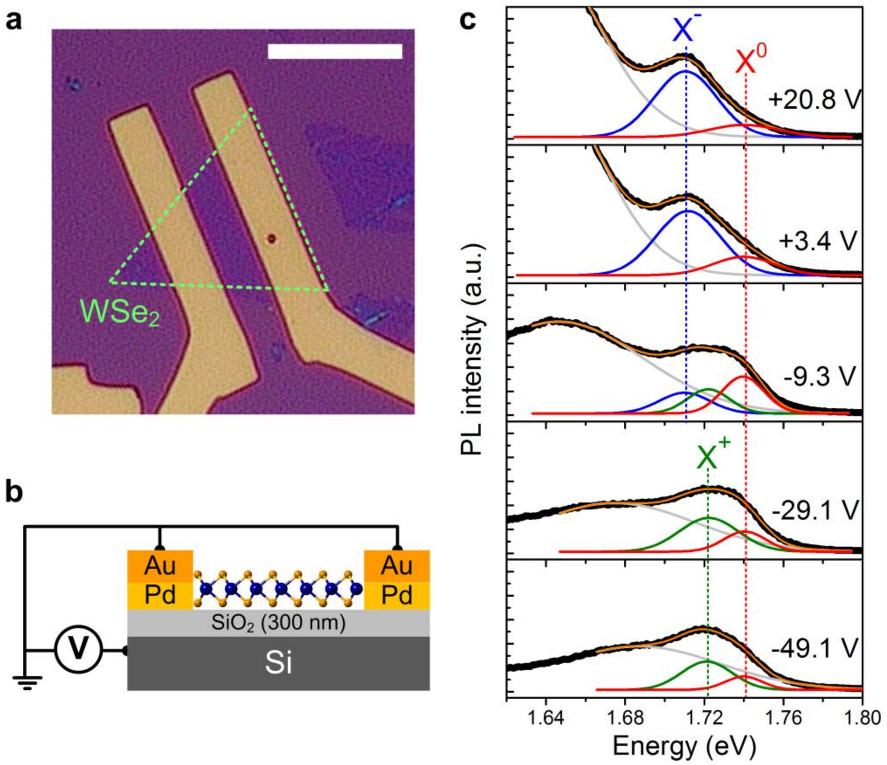

Supplementary Figure 2 | Gate-dependent PL spectra for monolayer WSe_2 on a back-gate device. (a) Optical microscopy image for a CVD-grown monolayer WSe_2 triangle on a 300-nm-SiO_2/Si substrate with Au(30 nm)/Pt(10 nm) metals as surface electrodes. The CVD-grown WSe_2 monolayers were transferred onto the SiO_2/Si substrate using a poly(methyl methacrylate) (PMMA) film as a supporting layer. The scale bar is 10 μm. (**b**) Schematic of the back-gate device structure. (**c**) The PL spectra (black) were measured at 80 K under various back-gate voltages. The evolution of neutral exciton (X^0) (red), positive trion (X^+) (green) and negative trion (X^-) (blue) peaks with the back-gate voltage can be deconvoluted by multiple Gaussian fitting. The binding energy for the positive (negative) trion is ~20±2 meV (~29±2 meV).

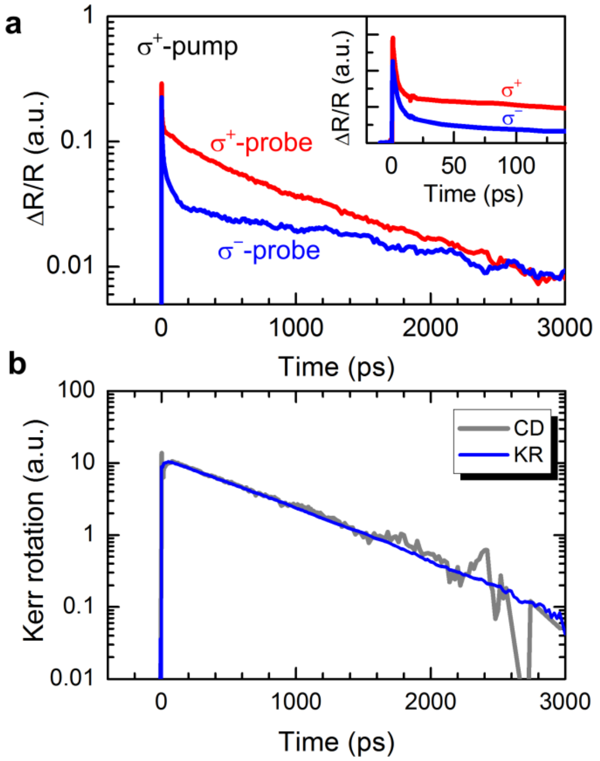

Supplementary Figure 3 | A comparison between Kerr rotation and helicity-resolved transient reflection. (a) The reflection changes $\Delta R/R$ probed by σ^+ (red curve) and σ^- (blue curve) pulses at 1.719 eV using σ^+ pumping at 1.731 eV. The time-average powers of the pump and probe beams are 500 and 100 μW, respectively, the same as that used in TRKR measurements. The reflection changes probed by different helicities show different decay dynamics and exhibit a strong circular dichroism (CD). The inset shows $\Delta R/R$ in a shorter timescale. (**b**) A comparison between the measured Kerr rotation (KR, blue curve) and CD (gray curve) using same experimental conditions. The temporal evolution of CD is obtained by taking the difference between $\Delta R/R$ traces probed by σ^+ and σ^- pulses.

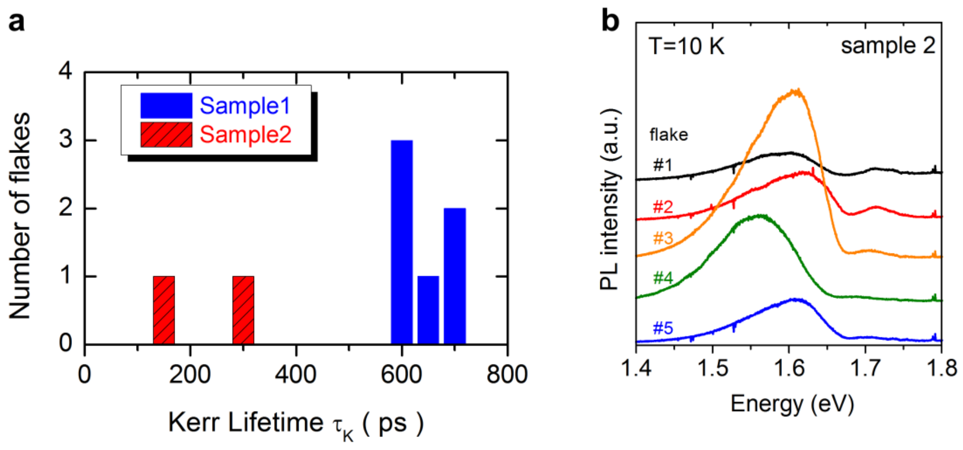

Supplementary Figure 4 | Distribution of measured Kerr lifetime. (a) Statistics of measured Kerr lifetime τ_K from different monolayer flakes on two samples. For sample 1, the Kerr lifetime is distributed in the range of 600-700 ps and varies only slightly from one to another. For sample 2, the Kerr lifetimes are significantly shorter and more scattered. For clarity, we only picked two data points. Our measurements indicated that the Kerr lifetime is sensitive to sample quality. The experimental data presented in main text are obtained from sample 1. **(b)** PL spectra of different monolayer flakes on sample 2.

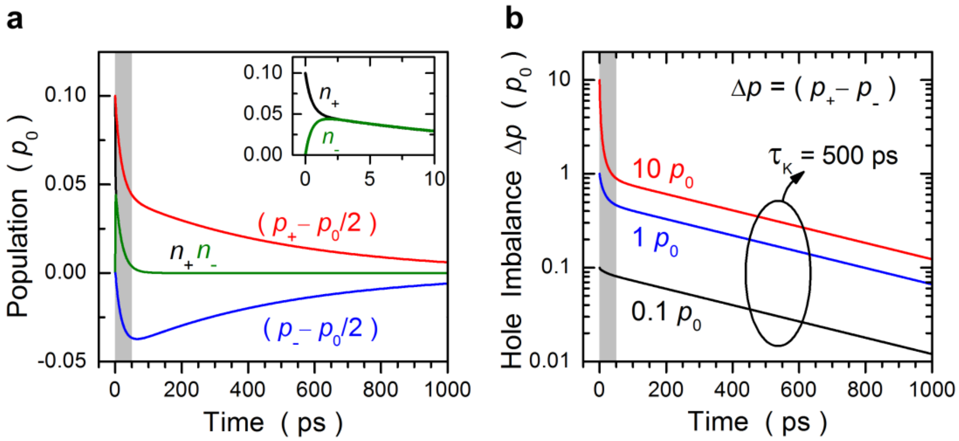

Supplementary Figure 5 | Temporal evolution of carrier population. (**a**) The calculated evolutions of the electron and hole populations in both valleys according to the rate equations considering separate depolarization of valley electrons and holes shown in Supplementary note 1. We assumed $\tau = 10\,\text{ps}$, $\tau_{\text{v,e}} = 0.5\,\text{ps}$, $\tau_{\text{v,h}} = 500\,\text{ps}$, and an initial photogenerated electron (hole) density of $n = p = 0.1p_0$. The inset shows the depolarization of valley electrons in a shorter time scale. (**b**) The evolutions of hole population imbalance $\Delta p = (p^+ - p^-)$ with different initial photocarrier densities: $n = p = 0.1p_0$, p_0 and $10p_0$.

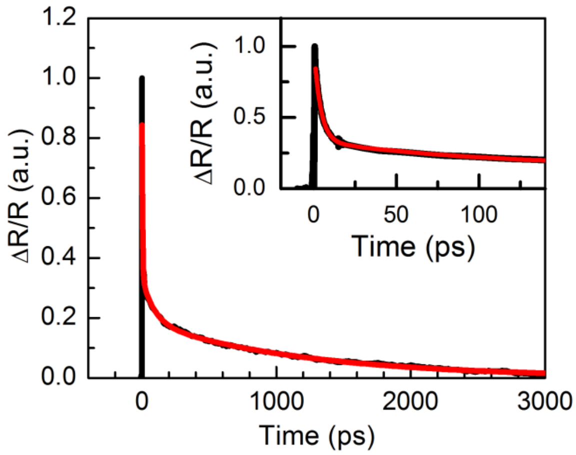

Supplementary Figure 6 | Pump-probe transient reflection. The reflection changes $\Delta R/R$ measured at T= 10 K by using linearly polarized probe pulses at 1.719 eV and pump pulses at 1.731 eV. The pump and probe beams are 500 and 100 μW, respectively, the same as that used in TRKR measurements. The inset shows $\Delta R/R$ in a shorter timescale. The red line is the fitting curve of a triple exponential decay function: $Ae^{-t/\tau_1}+Be^{-t/\tau_2}+Ce^{-t/\tau_3}$. The resulting time constants (relative weightings) are: $\tau_1=4.1$ ps (0.66), $\tau_2=89$ ps (0.15) and $\tau_3=1.2$ ns (0.19).

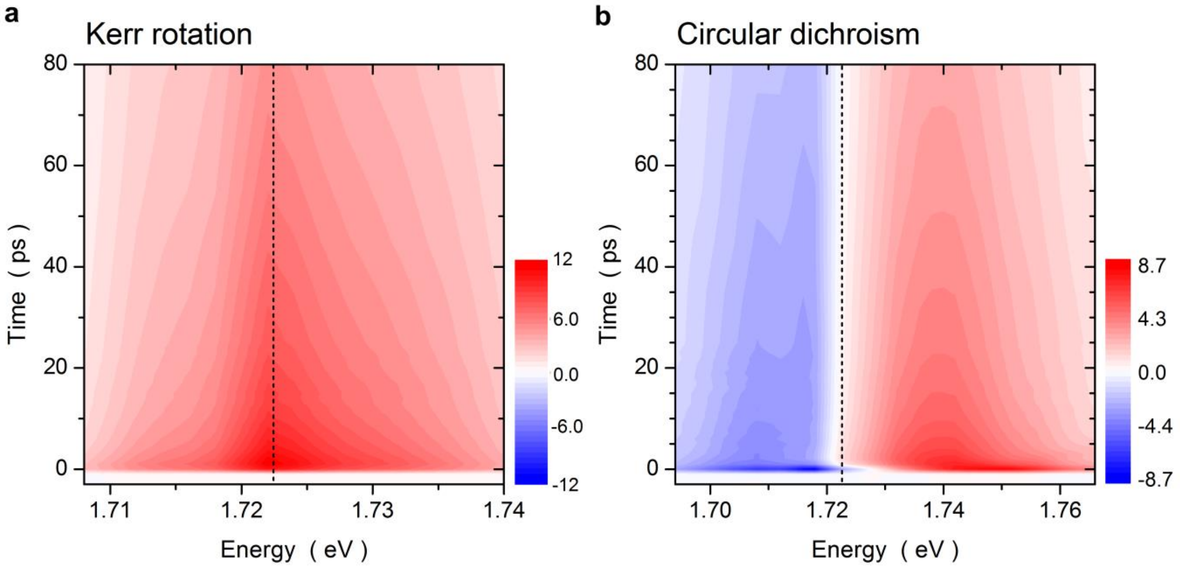

Supplementary Figure 7 | Kerr rotation and circular dichroism. Spectral responses of **(a)** Kerr rotation and **(b)** circular dichroism for monolayer WSe_2 on another sample. The experimental conditions are the same as that used in the main text. The Kerr rotation (circular dichroism) shows an absorptive (dispersive) spectral response near the trion resonance, indicating that the valley holes predominately affect the resonant energy or broadening of transitions in different valleys.

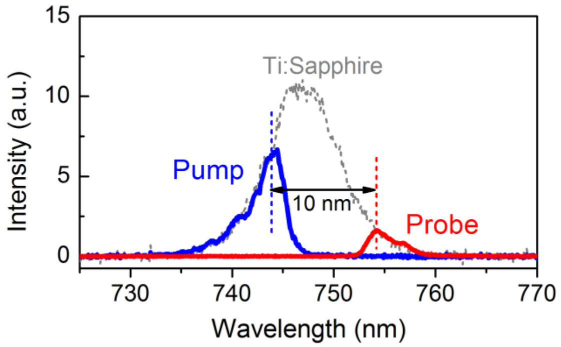

Supplementary Figure 8 | Typical spectra for the filtered pump and probe beams. The wavelengths of the pump and the probe pulses were tuned individually by two edge-pass filters. We adjust the tilted angles of the two filters, such that the pump energy is always ~12 meV higher than the probe. The well-separated pump and probe wavelengths facilitate us to block the pump beam scattering by another long-pass filter before entering the polarization bridge.

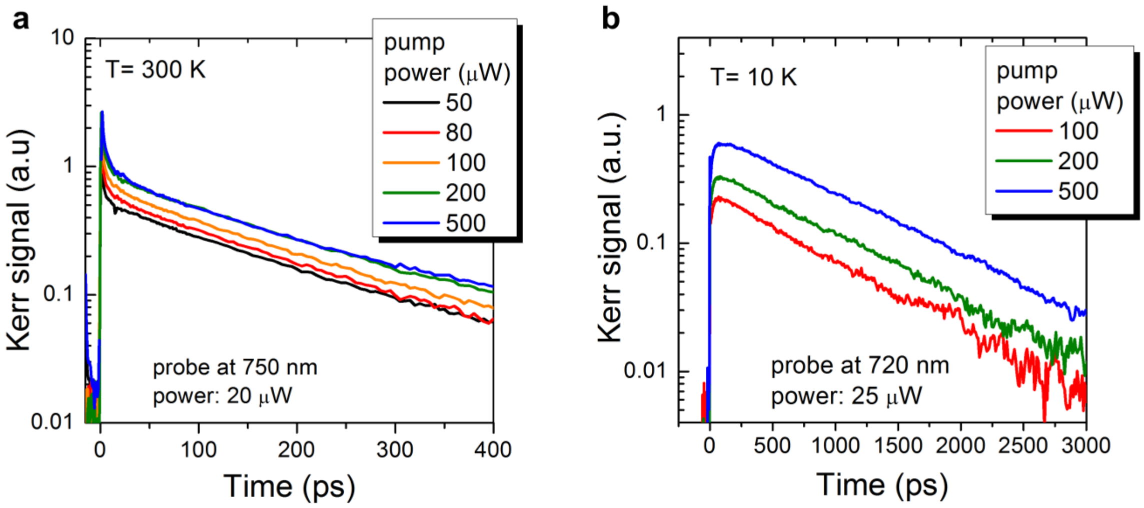

Supplementary Figure 9 | Pump power dependence of TRKR measurements. TRKR traces measured at **(a)** *T*= 300 K and **(b)** *T*= 10 K under different pump powers. The long-lived Kerr signals basically show the same decay dynamics for pump powers in the range of 50-500 μW.

Supplementary Note 1: Rate equation model

(1) Exciton valley dynamics

After creating excitons by a laser pulse at $t = 0$, the evolution of exciton population N_+ (N_-) in the $+\mathrm{K}$ ($-\mathrm{K}$) valley can be described by the following rate equations (Fig. 1a):

$$\begin{cases} \dfrac{dN_+}{dt} = -\dfrac{N_+}{\tau} - \dfrac{(N_+ - N_-)}{2\tau_\mathrm{v}} \\ \dfrac{dN_-}{dt} = -\dfrac{N_-}{\tau} - \dfrac{(N_- - N_+)}{2\tau_\mathrm{v}} \end{cases}, \tag{1}$$

where τ is the exciton recombination lifetime, and τ_v is the exciton valley lifetime, which is related to the intervalley scattering time τ_IS by $\tau_\mathrm{v} = \tau_\mathrm{IS}/2$. The temporal evolution of exciton population imbalance is given by

$$\Delta N(t) \equiv N_+(t) - N_-(t) = \Delta N(0)\exp(-t/\tau_\mathrm{K}), \tag{2}$$

where $\tau_\mathrm{K}^{-1} = \tau^{-1} + \tau_\mathrm{v}^{-1}$. If the intervalley scattering is very efficient ($\tau_\mathrm{v} << \tau$), then we have $\tau_\mathrm{K} \approx \tau_\mathrm{v}$. On the other hand, if the intervalley scattering is very slow ($\tau_\mathrm{v} >> \tau$), we have $\tau_\mathrm{K} \approx \tau$.

(2) Electron and hole valley dynamics

Now we consider separate depolarization of valley electrons and holes. The rate equations are:

$$\begin{cases} \dfrac{dn_+}{dt} = -\dfrac{n_+ p_+}{\tau} - \dfrac{(n_+ - n_-)}{2\tau_\mathrm{v,e}} \\ \dfrac{dp_+}{dt} = -\dfrac{n_+ p_+}{\tau} - \dfrac{(p_+ - p_-)}{2\tau_\mathrm{v,h}} \\ \dfrac{dn_-}{dt} = -\dfrac{n_- p_-}{\tau} - \dfrac{(n_- - n_+)}{2\tau_\mathrm{v,e}} \\ \dfrac{dp_-}{dt} = -\dfrac{n_- p_-}{\tau} - \dfrac{(p_- - p_+)}{2\tau_\mathrm{v,h}} \end{cases} \tag{3}$$

where $n_{+/-}$ ($p_{+/-}$) is the electron (hole) population in the $\pm\mathrm{K}$ valley, and $\tau_\mathrm{v,e}$ ($\tau_\mathrm{v,h}$) is the valley lifetime of electron (hole). Consider the initial carrier populations for p-doped samples:

$$\begin{cases} n_+(0) = n \\ p_+(0) = p + p_0/2 \\ n_-(0) = 0 \\ p_-(0) = p_0/2 \end{cases} \tag{4}$$

where n (p) are the photogenerated electron (hole) population (by σ^+ laser), and p_0 is the population of resident hole, which is presumably equally occupied ($p_0/2$) in both valleys at t =0.

Supplementary Fig. 5a shows the evolution of carrier populations, where we assumed $\tau = 10\,\mathrm{ps}$, $\tau_{\mathrm{v,e}} = 0.5\,\mathrm{ps}$, $\tau_{\mathrm{v,h}} = 500\,\mathrm{ps}$, and an initial photogenerated electron (hole) density of $n = p = 0.1 p_0$. Due to the short electron valley lifetime, electrons get depolarized first and become equally occupied in both valleys within a few picoseconds (inset in Supplementary Fig. 5a). After the carrier recombination in both $+\mathrm{K}$ and $-\mathrm{K}$ valleys (gray region), the holes become valley polarized. In Supplementary Fig. 9b, we depicts the evolution of hole population imbalance $\Delta p(t) = p_+ - p_-$ for different initial photocarrier densities. After the carrier recombination, $\Delta p(t)$ exhibits single-exponential decay with a lifetime equal to the hole valley lifetime ($\tau_{\mathrm{K}} = \tau_{\mathrm{v,h}} = 500\,\mathrm{ps}$). Under higher initial photoexcited carrier densities, an initial depolarization follows the carrier recombination.

Supplementary Note 2: Kerr rotation and circular dichroism

The spectral responses of the Kerr rotation $\theta(\omega)$ and the circular dichroism $CD(\omega)$ are related to the real and imaginary parts of the complex refractive index $\tilde{n}(\omega) = n(\omega) + i\kappa(\omega)$. Considering a Lorentz oscillator, the near-resonance spectral response of $n(\omega)$ is dispersive (i.e., with positive and negative wings around the resonance), while $\kappa(\omega)$ is absorptive (i.e., with a peak at the resonance). The spectral responses of $\theta(\omega)$ and $CD(\omega)$ are related to $n(\omega)$ and $\kappa(\omega)$ by:

$$\theta(\omega) = \frac{\omega d}{2c}[n_+(\omega) - n_-(\omega)], \qquad (5)$$

$$CD(\omega) = \frac{\omega d}{2c}[\kappa_+(\omega) - \kappa_-(\omega)], \qquad (6)$$

where d is the material thickness, c is the speed of light, and the subscripts (±) stands for light with different helicities ($\sigma^{\pm}$), which couples to the optical transitions in different valleys (±K). Therefore, the spectral responses of $\theta(\omega)$ and $CD(\omega)$ depend on how the valley carriers affects the exciton/trion resonances in different valleys. These effects include phase-space filling[1], screening of the Coulomb interaction[1], and bandgap renormalization[2], which would reduce the transition strength, or cause the transition to shift and broaden. If the population imbalance affects predominantly the transition strength, the spectral response of $\theta(\omega)$ [$CD(\omega)$] will be dispersive (absorptive). On the other hand, if the population imbalance mainly affects the resonant energy or broadening, $\theta(\omega)$ [$CD(\omega)$] becomes absorptive (dispersive).

We have measured spectral responses of $\theta(\omega)$ and $CD(\omega)$ for another sample at T= 10 K, as shown in Supplementary Fig. 6. In this experiment, we used the same experimental conditions as that described in the main text. The experimental set up for CD measurements is the same as that for TRKR, except that a quarter waveplate is inserted between the half waveplate and the polarization beam splitter (Fig. 2a). The observed absorptive $\theta(\omega)$ and dispersive $CD(\omega)$ spectral responses indicate that the valley holes predominately affect resonant energy or broadening of transitions in different valleys. In this sample, the resonance occur at 1.722 eV, which is close to the trion resonance at 1.719 for sample 1.

Supplementary References

1. Schmitt-Rink, S., Chemla, D. S. & Miller, D. A. B. Theory of Transient Excitonic Optical Nonlinearities in Semiconductor Quantum-Well Structures. *Phys. Rev. B* **32**, 6601-6609 (1985).

2. Kleinman, D. A. & Miller, R. C. Band-Gap Renormalization in Semiconductor Quantum Wells Containing Carriers. *Phys. Rev. B* **32**, 2266-2272 (1985).
